# Assessment of candidate ocular biomarkers of ageing in a South African adult population: Relationship with chronological age and systemic biomarkers^☆^

**DOI:** 10.1016/j.mad.2013.05.002

**Published:** 2013-07

**Authors:** Sophia Pathai, Clare E. Gilbert, Stephen D. Lawn, Helen A. Weiss, Tunde Peto, Colin Cook, Tien Y. Wong, Paul G. Shiels

**Affiliations:** a International Centre for Eye Health, Department of Clinical Research, Faculty of Infectious and Tropical Diseases, London School of Hygiene & Tropical Medicine (LSHTM), Keppel Street, London WC1E 7HT, UK; b Desmond Tutu HIV Centre, Institute of Infectious Diseases and Molecular Medicine, Faculty of Health Sciences, University of Cape Town, Anzio Road, Observatory 7925, Cape Town, South Africa; c Department of Clinical Research, Faculty of Infectious and Tropical Diseases, LSHTM, Keppel Street, London WC1E 7HT, UK; d MRC Tropical Epidemiology Group, Faculty of Epidemiology and Population Health, LSHTM, Keppel Street, London WC1E 7HT, UK; e NIHR Biomedical Research Centre for Ophthalmology, Moorfields Eye Hospital NHS Foundation Trust and UCL Institute of Ophthalmology, 162 City Road, London EC1V 2PD, UK; f Department of Ophthalmology, Groote Schuur Hospital, University of Cape Town, Observatory 7925, Cape Town, South Africa; g Singapore Eye Research Institute, National University of Singapore, 11 Third Hospital Avenue, Singapore 168751, Singapore; h Institute of Cancer Sciences, College of Medical, Veterinary & Life Sciences, University of Glasgow, Glasgow, UK

**Keywords:** Telomeres, CDKN2A, Lens density, Retinal vessel calibre, Corneal endothelium, Retinal nerve fibre layer, Frailty

## Abstract

Certain anatomic and functional parameters of the eye change with increasing chronological age. They may, therefore, serve as potential biomarkers of ageing. We investigated associations between four such ocular parameters (lens density, retinal vessel calibre, corneal endothelial cells and retinal nerve fibre layer thickness) and two ‘cellular’ biomarkers of ageing (leukocyte telomere length and CDKN2A expression), with frailty (a clinical correlate of biological ageing) in a population of South African adults. All ocular parameters revealed an association with either telomere length or CDKN2A expression. However, lens density was most strongly correlated with age, increased CDKN2A expression, and with frailty (*p* = 0.05 and 0.03, respectively). Narrow retinal arteriolar diameter, associated with increased chronological age, was also associated with increased CDK2NA expression (0.42 vs. 0.31, *p* = 0.02) but not with frailty. Ocular parameters may aid in determining biological age, warranting investigation in longitudinal studies.

## Highlights

• Structural parameters of the eye change with increasing chronological age.
• Ocular age-related parameters may serve as biomarkers of aging.
• Relevant ocular parameters include retinal vessel calibre and lens density.

## 1 Introduction

There is substantial variation in the health and functional status of older populations in many developing countries as well as in developed countries (Lloyd-Sherlock et al., 2012). The reasons for these variations are poorly understood, highlighting the need for translational age-related research within a global context (Salomon et al., 2013; Wang et al., 2013). Chronological age is an imprecise measure of biological ageing, due to inter-individual differences in rates of ageing. The disconnection between chronological age and lifespan has led to a search for effective and validated biomarkers of ageing (BoA), defined as “biological parameters of an organism that either alone or in some multivariate composite will better predict functional capability at some late age, than will chronological age” (Baker and Sprott, 1988).

It is acknowledged that many age-related chronic diseases such as cardiovascular disease and Alzheimer’s disease share common pathways of early dysregulation, and that the development of markers and diagnostic techniques is fundamental to understanding healthy biological ageing and thus these diseases (Franco et al., 2007). The need for research on how healthy ageing can be achieved in the context of life-time trajectories has led to concept of the ‘Healthy Ageing Phenotype’ (Franco et al., 2009). With demonstrable molecular, epigenetic and clinical correlates of ageing, the eye may be a model system for validating potential biomarkers (Pathai et al., 2013b).

The unique access to and visibility of ocular tissues and range of visual functions permits investigation of a wide variety of physiological and pathological mechanisms. Many age-related ocular changes also have systemic associations or correlates of ageing in other end-organs or body systems but may be easier and less invasive to measure in the eye (Table 1). For example, changes in the lens, which has an extremely high protein content, may reflect systemic changes in protein structure and function in other organs (Truscott, 2010, 2011; Wormstone and Wride, 2011). Corneal endothelial cell parameters, lens density, retinal vessel calibre and thickness of the retinal nerve fibre layer (RNFL) are ocular parameters that vary with age that can be objectively and non-invasively imaged and assessed.

Ideally, proposed ocular biomarkers should be assessed in relation to established and validated BoA at a clinical or cellular level. Only two validated BoA, telomere length (TL) and CDKN2A expression, have so far been found to satisfy the majority of the criteria proposed by Baker and Sprott (1988). Telomeres are nucleoprotein complexes at the ends of eukaryotic chromosomes. Their DNA component shortens with somatic cell division and upon reaching a critically short length, a DNA damage signal leads to growth cycle arrest, resulting in replicative senescence (Saretzki and Von Zglinicki, 2002; von Zglinicki, 2002). Telomere shortening is associated with increasing chronological age and several pathologies, including cardiovascular disease (Starr et al., 2007) and renal dysfunction (Carrero et al., 2008). TL may be useful as a composite measure of healthy ageing, but not as a BoA when used in isolation (Der et al., 2012; von Zglinicki, 2012). Expression levels of the cell cycle regulator CDKN2A may represent a more robust BoA (Shiels, 2010). CDKN2A acts as a tumour suppressor and maintains cells in a state of growth arrest, both in replicative and stress induced-senescence. Increasing levels of CDKN2A transcriptional expression occur with increasing age and decreasing function of solid organs and peripheral blood leucocytes (PBLs) (Koppelstaetter et al., 2008; Krishnamurthy et al., 2004; Liu et al., 2009; McGlynn et al., 2009). However, there are limited data on how these parameters correlate with measures of physical frailty (Woo et al., 2008), a functional state characterised by an increased risk of multiple pathologies, low physical activity and slow motor performance (Fried et al., 2001). Frailty predicts cognitive and physical decline and is associated with an increased risk of morbidity and mortality, and may therefore act as a ‘clinical’ biomarker of ageing (Fried et al., 2001).

There are few data on biological ageing in sub-Saharan Africa, a region where the population of elderly people is rapidly expanding, and where the incidence of age-related non-communicable diseases is steadily increasing (Marquez and Farrington, 2012). The aim of this study was to investigate the association of a variety of ocular candidate BoA with ‘systemic’ BoA and frailty status in a South African adult population.

## 2 Methods

### 2.1 Study population

Individuals aged ≥30 years from an HIV prevention trials site in a township community of Cape Town, South Africa (Emavundleni Centre, Crossroads) were recruited as HIV-seronegative controls as part a case–control study investigating HIV and ageing (Pathai et al., 2012, 2013a). Socio-demographic information and medical history were obtained by interviewing participants in their first language (Xhosa or English). Data collected included factors known to affect ageing (e.g. UV exposure, smoking history). All participants underwent a full ophthalmic examination including measurement of visual acuity, evaluation by slit lamp microscopy and indirect ophthalmoscopy.

The study was approved by the Ethics Committees of the London School of Hygiene and Tropical Medicine and the University of Cape Town Faculty of Health Sciences, and adhered to the tenets of the Declaration of Helsinki. Written informed consent was obtained from all participants.

### 2.2 Anthropometry, blood pressure and physical function including frailty assessment

Blood pressure (BP) was measured with a digital sphygmomanometer. Mean arterial blood pressure (MABP) was defined as two-thirds of the diastolic plus one-third of the systolic BP (Wong et al., 2003). Hypertension was defined as a systolic BP of 140 mmHg or higher, diastolic BP of 90 mmHg or higher, or the combination of self-reported high BP diagnosis and the use of anti-hypertensive medications (Wong et al., 2005). Body mass index (BMI) was defined as weight (in kilograms)/height^2^.

Physical frailty was defined by the presence of ≥3 of 5 criteria: (i) unintentional weight loss (self reported and verified from clinic records where possible), (ii) self-reported low physical activity, (iii) self-reported exhaustion, (iv) weak grip strength and (v) slow walking time. Pre-frailty was defined as the presence of one or two of these criteria. Detailed information is available in the Supplementary Methods.

### 2.3 Blood-based biomarkers

#### 2.3.1 DNA/RNA extraction

DNA was extracted from PBLs using the Maxwell™ Automated Purification System according to manufacturer’s instructions (Promega, USA). DNA concentration and purity were quantified by Nanodrop Spectrophotometer (ThermoFisher Scientific, USA). RNA was extracted using Trizol reagent (Invitrogen, UK) following manufacturer’s guidelines. DNA/RNA extraction was performed in Cape Town and samples shipped on dry ice to the University of Glasgow.

#### 2.3.2 Telomere length determination

Telomere lengths were determined by QPCR following the method of Cawthon (Cawthon, 2002). Telomere length determination was performed blindly using a Roche Light Cycler LC480. Briefly, telomere length analyses were performed in triplicate for each sample, using a single-copy gene amplicon primer set (acidic ribosomal phosphoprotein, 36B4) and a telomere-specific amplicon primer set (Koppelstaetter et al., 2008). Refer to Supplementary Methods for further detail.

#### 2.3.3 CDKN2A expression determination

Relative quantitative real-time PCR (qRT-PCR) was used to estimate mRNA levels corresponding to the candidate senescence associated gene – CDKN2A. Expression levels were measured against a reference hypoxanthine phosphoribosyltransferase (HPRT) housekeeping gene on an ABI Prism(R) 7500 Sequence Detection System. Sequences of human TaqMan™ Primer/Probe sets were designed by Primer Express algorithm (Applied Biosystems, Austin, TX, USA). The comparative threshold cycle method (ΔΔCT) (Livak and Schmittgen, 2001) was employed to quantify relative gene expression.

### 2.4 Ocular biomarkers

The following four ocular parameters were selected (Table 1). Detailed methods are supplied in the Supplementary Methods.

(i) *Lens density*: Pentacam imageing (Oculus, Wetzlar, Germany) was used to obtain “Scheimpflug images” of the lens and to obtain an objective estimate of lens density on a continuous scale. Lens density increases with increasing chronological age.
(ii) *Retinal vessel calibre*: Participants had stereoscopic 30̊ colour retinal photographs taken under pharmacological pupil dilation with a fundus camera (CF-2; Canon Inc., Tokyo, Japan). Vessel calibre indices were determined in a semi-automated manner using the IVAN computer program (Singapore Eye Research Institute, Singapore) and a standardized protocol described previously (Wong et al., 2004). Narrowing of retinal arterioles is associated with increasing chronological age (Leung et al., 2003; Wong et al., 2003).
(iii) *Corneal endothelial cell parameters*: A non-contact specular microscope was used (SP02, CSO; Florence, Italy). The operator focused and aligned a real-time image of the participant’s eye. Endothelial cell parameters were automatically calculated from this image by the microscope software. Endothelial cell density (ECD) decreases with age, whereas the change in cell size (coefficient of variation) increases with age. The proportion of cells with six sides (hexagonality index) decreases with age.
(iv) *Retinal nerve fibre layer* (*RNFL*): Measured using Spectral OCT/SLO optical coherence tomography (Opko/OTI Inc, Miami, FL) which uses a scanning laser diode of 830 nm to provide images of ocular microstructures. A peripapillary (around optic nerve head) protocol inbuilt in the software was used to determine the average and quadrant-specific RNFL thickness (superior, inferior, temporal and nasal). The RNFL becomes thinner with increasing age (Chi et al., 1995; Kanamori et al., 2003).

### 2.5 Statistical analysis

One eye was randomly selected for analysis. Where an eye was not available, for example due to trauma or corneal opacity, the contralateral eye was used. Analyses were performed using Stata 12 (Stata Corp., College Station, TX). Clinical and biological data were summarized as the median with interquartile range (IQR) or mean with standard error (SE), as appropriate. Analyses were conducted on log 10-transformed values of telomere length and mean CDKN2A expression to satisfy the assumption of normally distributed residuals. Results are displayed back-transformed to the original scale. Validation of the biomarkers was performed using linear regression models with age in years as a continuous or categorical variable. Ocular biomarker measurements were divided into quartiles. Univariable linear regression was performed to compare the quartiles of ocular parameters with mean telomere length and CDKN2A expression and frailty status respectively. Multivariable linear regression models were used to examine the relationships of telomere length, CDKN2A expression as the respective dependent variable with ocular biomarker quartiles and frailty status and explanatory variables (age group [30–39; 40–49; >50 years], gender, MABP; BMI, smoking, UV exposure) as independent variables. Marginal adjusted means for telomere length and CDKN2A expression were estimated at the mean value of covariates in the model. The Wald test was used to assess statistical significance of the association of each ocular parameter on systemic biomarker levels.

## 3 Results

### 3.1 Participant characteristics and biomarker distributions

256 participants underwent assessment. Their median age was 40 years (IQR: 35–49) and 25% (*n* = 64) were male. Characteristics of the participants by gender are given in Table 2. Women reported less alcohol consumption and cigarette use than men (*p* < 0.0001 for both). Men had a higher frequency of hypertension and had lower BMI (*p* = 0.01 and *p* < 0.0001, respectively). The number of participants providing data for each parameter varied, as not every participant was able to complete every ophthalmic test or had a blood sample available for analysis. Summary statistics for each biomarker, stratified by gender and age group are displayed in Table S1. For the majority of biomarkers, there was no evidence of gender differences; however for RNFL parameters, the average, inferior and temporal quadrants were thinner in men (*p* = 0.01, *p* = 0.0008 and *p* = 0.02, respectively).

### 3.2 Validation of blood-based and ocular biomarkers against chronological age

**Fig. 1:**
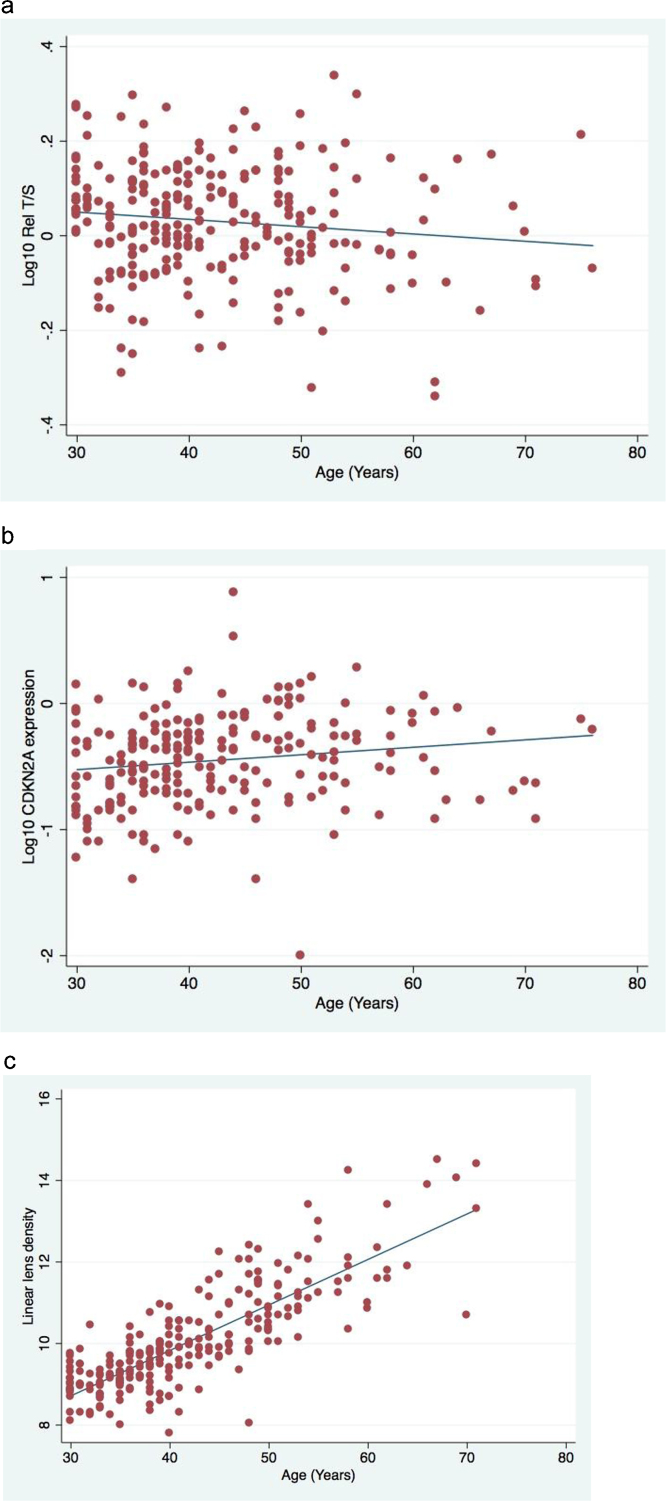
Scatter plots of biomarkers with chronological age. (a) Telomere length, *R*^2^ = 0.02; (b) CDKN2A expression, *R*^2^ = 0.02; (c) linear lens density, *R*^2^ = 0.67.

Blood-based and ocular parameters were validated against chronological age (Table S2). All parameters except retinal venular diameter and the RNFL nasal quadrant were related to chronological age and so these parameters were not analysed further. The association of telomere length, CDKN2A and linear lens density with chronological age is presented Fig. 1a–c. The association of the other prospective biomarkers is presented in Supplementary Fig. 1d–o. The *R*-squared values of the regressions against chronological age were highest for lens density parameters (linear lens density *R*^2^ = 0.67); other biomarkers including TL and CDKN2A had *R*^2^ values <0.10. All analyses thereafter were adjusted for age, gender and other possible confounding variables related to the parameter of interest.

### 3.3 Association of ocular parameters with blood-based biomarkers

Shorter TL was associated with decreasing endothelial cell density (*p*-trend = 0.08). CDKN2A expression was related to increased variation in endothelial cell size in a non-linear fashion (*p* = 0.05) (Table 3). The linear parameter of lens density was most informative (Table 3). CDKN2A expression increased with increased lens density (*p*-trend = 0.05); the 3-D average lens density parameter also displayed a similar trend (*p*-trend = 0.08).

Expression of CDKN2A was associated with changes in the calibre of retinal arterioles (*p* = 0.06) and in AVR (*p* = 0.03) (Table 3), however a linear trend was not detected. When arteriolar diameter was categorised as a binary variable, (‘thin’ or ‘thick’, i.e. either side of the median value) CDKN2A expression was greater in those with thin compared to thick retinal arterioles (0.42 vs. 0.31, *p* = 0.02). TL was not related to arteriolar diameter.

In view of the difference in RNFL thickness between genders (Table S1), data were analysed for RNFL by gender. After adjustment, there were no differences between genders (data not shown), thus data for both men and women combined are presented. Telomere length was informative for the superior quadrant of the RNFL (Table S3), with shorter telomere length associated with thinner RNFL (*p*-trend = 0.05).

### 3.4 Association of frailty status with blood-based and ocular biomarkers

There was a significant trend of increased TL with worsening frailty status (*p*-trend = 0.02) (Table 4). CDKN2A expression also increased with frailty status, however this trend was not statistically significant (*p*-trend = 0.12). Among the ocular biomarkers, lens density was the only parameter associated with frailty status, with increased linear lens density related to greater frailty status (*p*-trend = 0.03).

## 4 Discussion

In this study we compared several ocular parameters with established and validated systemic BoA (TL and CDKN2A expression) as well as frailty, a clinical correlate of ageing. Objective measurement of lens density was the most informative ocular biomarker, with greater lens density associated with increased CDKN2A expression and with increased frailty status. Retinal arteriolar narrowing was also associated with greater CDKN2A expression. In contrast, lower endothelial cell density and thinning of the RNFL were associated with shorter TL. These findings suggest that a range of structural features of the eye, which can be objectively imaged and measured, may reflect different physiological parameters of ageing. These ocular BoA may provide insights into biological age, ageing trajectories and a range of chronic systemic diseases.

Lens density parameters had the strongest association with chronological age compared to the other biomarkers and fulfil the Baker and Sprott criteria (Baker and Sprott, 1988). Lens density was the most informative ocular biomarker in that it was related to CDKN2A expression, a cellular biomarker of ageing, as well as with frailty status, a clinical correlate of systemic ageing. The human lens is considered an ideal tissue for studying macromolecular ageing, and physiological ageing in general, as biochemical mechanisms in lens proteins may reflect ageing processes elsewhere in the body (Eldred et al., 2011; Michael and Bron, 2011; Truscott, 2010, 2011; Truscott and Zhu, 2010; Wormstone and Wride, 2011). Epidemiological research has demonstrated that individuals with cataracts have a significantly higher mortality rate than those without, even after adjusting for known confounders (Wang et al., 2001; West et al., 2000). Crystallins represent the major structural proteins of the lens and are responsible for the refractive power of the lens (Horwitz, 2000). In other tissues crystallins are also involved in several cellular pathways involving the stress response, apoptosis and cell survival at a systemic and ocular level (Andley, 2008). Thus, crystallins are not only involved in regulatory roles within the eye but also play important roles in several other organs, leading to the suggestion that cataract is a ‘bio-indicator’ for less obvious, more severe age-related disorders (Graw, 2009). The concept of ‘lens transparency’ as a biomarker of ageing has already been described (Sanders et al., 2011), and clinical diagnosis of cataract is associated with leukocyte TL. However clinical cataract is generally diagnosed in later years of life, whereas Scheimpflug imageing of lens density provides objective measurements at any given age. Evaluation of lens density across the age spectrum would be essential to evaluate fully the usefulness of the lens as a biomarker of ageing, and testing whether lens transparency is a predictor of mortality or longevity would provide the strongest evidence. However, this approach may be limited in well-resourced settings where surgical extraction of the lens often occurs with minimal lens opacities, rendering the lens unavailable for assessment. However, lens density measurement from early ages until lens extraction may still provide insight into healthy ageing trajectories.

The retina represents a unique location where the microvasculature can be directly and non-invasively visualised. The technique of semi-automated software applied to digital retinal photographs (Wong et al., 2004) is established as a valid and efficient biomarker of systemic vascular disease (Ikram et al., 2006; Wong, 2006; Wong et al., 2002). Retinal vascular calibre is considered a structural marker of vascular pathology reflecting the interplay of systemic, environmental and genetic factors (Sun et al., 2009). The strong association between increasing age and narrowed retinal vessels has been demonstrated in several study populations (Leung, 2003; Wong, 2003). Small reductions in retinal arteriolar calibre are associated with clinically relevant changes in blood pressure, e.g. a 10 mmHg increase in systolic BP is associated with a 1.1 μm reduction in arteriolar calibre (Ikram et al., 2004). We found that retinal arteriolar narrowing was associated with increased CDKN2A expression, thus the retinal microvasculature reflect senescent microvascular changes. However, retinal vessels can also be affected by systemic pathology e.g. rheumatoid arthritis, smoking and inflammatory diseases (Ikram et al., 2004; Klein et al., 2006; Van Doornum et al., 2011) and may be manifest as a change in retinal vessel calibre. This could affect the measurement of ‘true’ biological ageing, therefore the lens might be a better model and biomarker of ageing as it is less susceptible to systemic pathology, and therefore representing a true biomarker of ageing and not disease (Simm et al., 2008).

Corneal endothelial cells change morphology and assume an ‘aged phenotype’ in several chronic systemic diseases (e.g. renal failure, diabetes) (Larsson et al., 1996; Ohguro et al., 1999) suggesting that they may be useful in assessing cellular dynamics of ageing, particularly as measurement is objective and non-invasive. Reduction in the proliferative capacity of corneal endothelial cells is partly mediated by an age-related increase in expression of CDKN2A that functions to hold a cell in a state of growth arrest (Wang et al., 2012). Increased CDKN2A expression was noted in those with the lowest endothelial cell density, however this trend was not significant. In contrast, we found a trend of decreasing endothelial cell density with shorter TL. Evaluation of corneal endothelial cells via specular microscopy may provide a unique way of measuring biological ageing at a cellular level.

Thinning of the RNFL is associated with older age (Chi et al., 1995; Kanamori et al., 2003), manifest functionally as deficits in colour vision and contrast sensitivity. Our findings of thinner superior RNFL associated with shorter TL are in alignment with data from individuals with age-related neurocognitive disease. Thinning of the superior RNFL in Alzheimer’s disease has been observed (Lu et al., 2010; Paquet et al., 2007) and similar findings have been noted in Parkinson’s disease and spinocerebellar ataxias (Hajee et al., 2009; Pula et al., 2011). OCT measurement of the RNFL is a quick non-invasive procedure, of importance for patients with cognitive impairment. The retrograde loss of nerve fibre layer tissue in the retina and optic nerve may be an early biomarker of Alzheimer’s disease, and possibly the earliest sign of disease, prior to damage to the hippocampal region that impacts memory (Valenti, 2011). Thus RNFL analysis may be best suited in detection of early neurocognitive decline as a marker of ‘neurobiological ageing’.

In relation to frailty and TL, a study (Woo et al., 2008) showed no correlation between TL and frailty index. Indeed, women had higher fraility scores and longer TLs. We also detected longer TL with increasing frailty status which is not intuitive, as shorter telomeres would be expected. One possible explanation for this, is that induction of stress induced premature senescence (SIPS) (Shay and Wright, 2000), leads to acute growth arrest (in contrast to gradual replicative senescence). This may occur in frailty by a non-quantified factor (e.g. genetics, environment, lifestyle) acting on the affected cell population. Our finding that CDKN2A expression does not follow a similar trend is supportive of such a hypothesis. This would then be expected to result in longer TLs in cells under SIPS contributing to frailty. An alternative explanation is a ‘survivor effect’ i.e. individuals with poor biological ageing may die earlier, thus participants comprise survivors who have different biological characteristic to non-survivors, accounting for frail ‘survivors’ having longer telomeres. The relationship between the functional phenotype of frailty (reflecting changes in multi-organ systems) and cellular senescence represents two extremes of biological ageing likely influenced by several external factors, thus a well-defined relationship between these parameters maybe unlikely in any case. Lens density was the only ocular parameter to be associated with frailty, reinforcing its potential role as a biomarker of ageing with associations with cellular BoA as well as the clinical presentation of frailty.

This study has some limitations. Study participants were matched by age, gender and socio-economic status to HIV-infected individuals as part of a case–control study, and are not therefore representative of the general population in South Africa. For example, the gender composition of participants was three-quarters female, reflective of the HIV epidemic in Africa, but not representative of the South African population. There were also differences between genders (smoking, alcohol consumption) that may truly exist or may have been misclassified (e.g. misreporting true smoking habits) and this could have confounded associations of the ocular parameters with the other biomarkers. Participants were recruited from a community of considerable socio-economic depravation, and therefore likely to have been exposed to factors known to increase biological ageing such as high UV exposure from outdoor work. Therefore, our data might over-estimate associations related to ageing. Lastly, as study participants are of African ancestry, our results are generalizable to the African population.

In conclusion, our study suggests that the eye has a useful and valid contribution to make in the assessment of biological age. The non-invasive and objective nature of the techniques is an added benefit. Our data suggest that assessment of retinal vessel calibre and lens density may be most informative. Further studies could involve development of an ‘ocular ageing index’ using ocular parameters to predict not only visual morbidity (visual impairment/blindness), but also systemic morbidity and mortality. RNFL parameters may be useful in developing an index for age-related neurocognitive decline, whereas endothelial cell parameters may aid in understanding cellular mechanisms of senescence. In resource-constrained settings access to facilities and personnel capable of measuring biomarkers extracted from PBLs is likely to be limited. Thus, ocular biomarkers in parallel with more easily measurable systemic biomarkers (e.g. frailty status, blood counts) to assess biological ageing may be more feasible in these environments. Finally, we have proposed a research agenda to further define and validate ocular biomarkers of ageing (Pathai et al., 2013b). Longitudinal studies in different populations are needed to assess how ocular parameters change over time in relation to blood-based biomarkers and to other candidate biomarkers that have been previously evaluated (Martin-Ruiz et al., 2011; Simm et al., 2008). These parameters could also be measured in longitudinal evaluation of the ‘healthy ageing phenotype’ from early adult life onwards to characterise the development of biological capital and ageing trajectories in terms of a ‘life course’ approach (Kuh, 2007) leading to an improvement in our understanding of how to achieve healthy ageing in societies with rapidly increasing ageing populations.

## Funding

This work was supported by a Wellcome Trust grant awarded to SP (Grant number: 090354/Z/09/Z). SDL is funded by the Wellcome Trust (Grant # 088590). TP is funded by NIHR BRC at MEH and IoO.

## Figures and Tables

**Table 1:** Biomarkers of aging, methods of measurement and the impact of aging.

Anatomical site	Parameter	Method of measurement	Age-related changes
Peripheral blood leukocytes	Telomere length (TL)	qPCR	TL shortens
	CDKN2A expression	qRT-PCR to estimate mRNA levels	Increased expression

Corneal endothelium	Endothelial cell density (ECD)	Specular microscopy	Decreased ECD
	Coefficient of variation (CV)		Increased CV
	Hexagonality index (Ex)		Decreased Ex
Lens	Lens opacity	Pentacam – lens densitometry	All increase
	Linear value		
	Peak		
	3D average		
Retina	Retinal nerve fibre layer (RNFL) thickness (average, superior, inferior, nasal, temporal quadrants)	Optical coherence tomography (OCT)	Thinner RNFL – all quadrants
	Retinal vessel calibre	Semi-automated retinal analysis software applied to fundus photographs	Reduced diameter of arterioles and arterio-venous ratio (AVR)

Systemic	Frailty status	Assessment of walking speed, grip strength, self-report of weight loss, exhaustion and low physical activity	Frailty status increases
	Non-frail (no criteria)		
	Pre-frail (1–2 criteria)		
	Frail: ≥3 of 5 criteria		

**Table 2:** Characteristics of study participants, n = 256.

Variable	Male (*N* = 64) *N* (%)	Female (*N* = 192) *N* (%)	*p*
Age (mean ± SE)	42.0 ± 1.00	42.8 ± 0.7	0.61
Median age, IQR	41 (35–48)	40 (35–49)	0.96

Age, years by group			
30–39	27 (42.2)	91 (47.4)	
40–49	28 (43.7)	56 (29.2)	
>50	9 (14.1)	45 (23.4)	0.07

Education			
Did not complete high school	15 (23.4)	32 (16.7)	
Completed high school	49 (76.6)	160 (83.3)	0.23

Income			
<ZAR1000/month	41 (64.1)	133 (69.3)	
>ZAR1000/month	23 (35.9)	59 (30.7)	0.44

Location of work			
Outdoors or grant-holder	44 (68.8)	140 (72.9)	
Indoors	20 (31.2)	52 (27.1)	0.52

Housing			
Formal	38 (59.4)	114 (59.4)	
Informal	26 (40.6)	78 (40.6)	0.99

Shared WC			
No	19 (29.7)	52 (27.1)	
Yes	45 (70.3)	140 (72.9)	0.69

Number of people in household			
Up to 5	40 (72.7)	125 (68.7)	
≥6	15 (27.3)	57 (31.3)	0.57

Smoking status			
Non-smoker	23 (35.9)	162 (83.4)	
Smoker; <10 years	16 (25.0)	17 (8.9)	
Smoker >10 years	25 (39.1)	13 (6.8)	<0.0001

Alcohol			
Nil	14 (21.9)	128 (66.7)	
<1 L/week	14 (21.9)	43 (22.4)	
>1 L/week	36 (56.2)	21 (10.9)	<0.0001

Hypertension			
No	36 (56.3)	141 (73.4)	
Yes	28 (43.7)	51 (25.6)	0.01

BMI			
<20	14 (21.9)	4 (2.1)	
20–24.9	29 (45.3)	17 (8.9)	
25.0–29.9	13 (20.3)	42 (21.9)	
>30	8 (12.5)	129 (67.2)	<0.0001

Tuberculosis status			
No history	52 (81.3)	173 (90.1)	
Previous history	12 (18.7)	18 (9.4)	
Current	0	1 (0.5)	0.12

**Table 3:** Association of ocular parameters with blood-based biomarkers.

Endothelial cell parameters	Telomere length			CDKN2A expression		
	*N*	Rel T/S	*p*	*N*	Mean	*p*
ECD^a^ quartiles cells/mm^2^						
1st (1952–2461)	59	1.06 (0.99–1.14)		47	0.42 (0.33–0.53)	
2nd (2466–2612)	59	1.03 (0.96–1.10)		52	0.32 (0.25–0.40)	
3rd (2617–2798)	58	1.10 (1.03–1.18)		54	0.36 (0.29–0.45)	
4th (2812–3391)	60	1.14 (1.06–1.22)	*p*-Trend 0.08	51	0.36 (0.29–0.46)	0.30

CV^b^ quartiles						
1st (26–32)	66	1.03 (0.97–1.10)		47	0.35 (0.28–0.38)	
2nd (33–35)	70	1.08 (0.99–1.15)		52	0.31 (0.25–0.38)	
3rd (36–38)	49	1.13 (1.04–1.22)		54	0.49 (0.38–0.64)	
4th (39–57)	51	1.10 (1.02–1.19)	0.33	51	0.36 (0.28–0.46)	0.05

Ex^c^ quartiles						
1st (24–46)	67	1.13 (1.06–1.20)		57	0.36 (0.29–0.45)	
2nd (47–50)	59	1.05 (0.98–1.13)		61	0.40 (0.32–0.51)	
3rd (51–54)	60	1.08 (1.00–1.15)		40	0.35 (0.28–0.44)	
4th (55–70)	50	1.06 (0.98–1.14)	0.50	46	0.33 (0.25–0.42)	0.66

**Lens density**^d^						
Linear quartiles						
1st (7.8–9.2)	62	1.01 (0.94–1.10)		51	0.29 (0.22–0.38)	
2nd (9.25–9.8)	59	1.08 (1.00–1.17)		53	0.31 (0.24–0.40)	
3rd (9.85–10.75)	58	1.14 (1.06–1.22)		50	0.37 (0.29–0.47)	
4th (10.8–14.5)	61	1.09 (0.99–1.19)	*p*-Trend 0.22	54	0.50 (0.37–0.68)	*p*-Trend 0.05

Peak quartiles						
1st (10.65–15.35)	61	0.93 (1.00–1.16)		57	0.36 (0.28–0.45)	
2nd (15.4–18.0)	58	1.07 (1.00–1.15)		50	0.33 (0.26–0.41)	
3rd (18.05–20.5)	61	1.10 (1.03–1.18)		51	0.37 (0.29–0.46)	
4th (20.6–52.1)	60	1.07 (0.99–1.15)	0.92	50	0.39 (0.30–0.51)	0.81

3D-average quartiles						
1st (8.3–9.1)	58	1.01 (0.92–1.09)		52	0.28 (0.21–0.37)	
2nd (9.15–9.75)	64	1.08 (1.00–1.16)		54	0.39 (0.31–0.50)	
3rd (9.8–10.85)	57	1.14 (1.06–1.23)		50	0.33 (0.26–0.43)	
4th (10.9–19.7)	61	1.10 (1.00–1.20)	*p*-Trend 0.16	52	0.46 (0.34–0.62)	*p*-Trend 0.08

**Retinal vessels**						
Retinal arteriolar quartiles (μm)						
1st (102.62–150.30)	57	1.05 (0.98–1.13)		48	0.40 (0.31–0.51)	
2nd (150.36–161.09)	59	1.13 (1.05–1.21)		50	0.43 (0.34–0.54)	
3rd (161.35–172.72)	60	1.02 (0.95–1.09)		55	0.28 (0.22–0.35)	
4th (172.74–209.71)	61	1.10 (1.03–1.19)	0.15	53	0.34 (0.27–0.44)	0.06

Endothelial parameters: adjusted for age, gender, smoking, UV exposure, income. Lens density: adjusted for age, gender, smoking, UV exposure. Vessels: adjusted for age, gender, smoking, BMI, hypertension and venular retinal calibre; 1st quartile denotes aged phenotype for all vessel parameters.

a Endothelial cell density –lowest quartile denotes aged phenotype.

b Coefficient of variation – i.e. difference in cell shape; highest quartile denotes aged phenotype.

c Hexagonality index – i.e. proportion of cells that are hexagonal; lowest quartile denotes aged phenotype.

d Measured on a continuous scale 0–100, 100 being an opaque (completely dense) lens; 4th quartile denotes aged phenotype for all lens parameters.

**Table 4:** Association of frailty with systemic and ocular biomarkers of aging.

Biomarker	Not-frail	Pre-frail	Frail	*p*
Telomere length (*N*) Rel T/S	89	127	34	
	1.01 (0.96–1.08)	1.09 (1.04–1.14)	1.17 (1.06–1.29)	*p*-Trend 0.02

CDKN2A (N) Mean expression	75	110	32	
	0.32 (0.26–0.39)	0.36 (0.31–0.42)	0.44 (0.32–0.60)	*p*-Trend 0.12

**Ocular parameter**				
Lens density (*N*) Scale: 0–100	92	123	31	P
Linear	9.94 (9.77–10.13)	10.10 (9.94–10.25)	10.38 (10.05–10.71)	*p*-Trend 0.03
Peak	18.82 (17.78–19.85)	18.68 (17.81–19.54)	18.86 (17.01–20.71)	0.97
3D-Average	10.02 (9.81–10.24)	10.14 (9.95–10.31)	10.31 (9.92–10.70)	*p*-Trend 0.22

Vessel calibre (*N*) μm	92	120	31	
CRAE	160.13 (156.56–163.70)	160.99 (157.97–164.01)	166.30 (159.87–172.72)	*p*-Trend 0.19
AVR	0.60 (0.58–0.61)	0.60 (0.58–0.61)	0.61 (0.58–0.64)	0.64

Endothelial cell parameters (*N*)	91	121	31	
ECD^a^ cells/mm^2^	2637 (2580–2695)	2587 (2539–2635)	2675 (2572–2779)	0.18
CV^b^	35.1 (34.1–36.1)	35.3 (34.4–36.1)	36.9 (35.1–38.8)	*p*-Trend 0.18
Ex^c^	49.9 (48.6–51.3)	50.3 (49.1–51.4)	47.6 (42.3–50.0)	0.15

**RNFL parameters (*****N*****) μm**				
Average	109.9 (106.7–113.2)	108.9 (105.6–110.6)	106.9 (101.4–112.4)	*p*-Trend 0.31
Superior	131.1 (126.2–136.1)	133.4 (129.3–137.4)	130.6 (121.5–139.6)	0.73
Inferior	137.6 (132.6–142.6)	138.2 (134.1–142.3)	137.3 (127.2–146.5)	0.97
Temporal	72.3 (69.1–75.4)	72.6 (70.1–75.2)	72.2 (66.4–77.9)	0.98

Adjusted for age, gender, socio-economic status, smoking, alcohol consumption and TB status.

a Endothelial cell density – lowest quartile denotes aged phenotype.

b CV: coefficient of variation – i.e. difference in cell shape; highest quartile denotes aged phenotype.

c Hexagonality index – i.e. proportion of cells that are hexagonal; lowest quartile denotes aged phenotype.
