# Supplementary material for: Assessment of candidate ocular biomarkers of ageing in a South African adult population: Relationship with chronological age and systemic biomarkers
Source: Mech Ageing Dev. 2013 Jul;134(7-8):338–45. doi: 10.1016/j.mad.2013.05.002 (PMC3710972; doi:10.1016/j.mad.2013.05.002)
Supplement: Supplementary file 1 [file mmc1.docx]

### Supplementary Methods

### Telomere length determination:

### Quality control parameters for the amplifications comprised a cut off of 0.15 for the standard deviation (SD) of the threshold cycle (Ct) for sample replicates. At a SD above 0.15 the sample was reanalysed. The average SD across plates was 0.05. Relative telomere length was estimated from Ct scores using the comparative Ct method after confirming that telomere and control gene assays yielded similar amplification efficiencies. This method determines the ratio of telomere repeat copy number to single copy gene number (T/S) ratio in experimental samples relative to a control sample DNA. This normalised T/S ratio was used as the estimate of relative telomere length (Relative T/S). The inter-assay variation was assessed by comparing the relative telomere estimates (T/S ratio) estimates across assays for the positive controls, assayed on every assay plate. The average inter-assay coefficient of variance was 0.6% for telomere length and 0.23% for 36B4.

**Frailty determination**

All of these five components described in the original phenotype by Fried et al ([Fried et al., 2001](#_ENREF_2)) were used to determine the presence of frailty. However, we used the proxy described by Önen et al ([Onen et al., 2009](#_ENREF_7)) for the physical activity measure (see Table 1 below for description). Grip strength of the dominant hand was measured three times using a grip dynamometer (Jamar Plus+ Digital Hand Dynamometer, Jamar, US). The average of three weight measurements was recorded in kilograms (kg) to one decimal point. Walking time was assessed using the method of Cesari et *a*l ([Cesari et al., 2005](#_ENREF_1)). The average of two trials (in m/s) was used for analysis. Participants were excluded from the determination of grip strength if they had pain or arthritis of the dominant hand, and excluded from the walking test if they had paralysis of an extremity or side of the body, or needed to use a walking aid.

**Lens density measurement:**

Following pupil dilatation with 1% tropicamide, two lens density measurements were made on each eye, and the mean value calculated. The instrument automatically calculated the quality and reliability of a captured image. If an image was found to be of poor quality (i.e. not ‘OK’ on the image quality specification), the measurement was repeated. Reproducibility of the lens density evaluation in two scans was performed for 50 eyes. Lens densitometry output values were extracted from the image captures in a masked fashion. Image section 90-270 degrees was used for the right eye and image section 270-90 degrees was used for the left eye ([Kirkwood et al., 2009](#_ENREF_4)). All of the densitometry metrics available from the scan (linear, peak and 3D) were analysed as they reflect different parameters of the lens. The lens density output was presented on a continuous scale from 0 (transparent) to 100 (fully opaque).

**Retinal vessel measurement**

All participants had stereoscopic 30 degree colour retinal photographs taken of both eyes under pharmacological pupil dilation with a fundus camera (model CF-2; Canon Inc., Tokyo, Japan). Images were centred on the optic disc. Vessel calibre indices were determined in a semi-automated manner using the IVAN computer program (Singapore Eye Research Institute, Singapore) using a standardized protocol described previously ([Wong et al., 2004](#_ENREF_10)). In summary, the 6 largest arterioles and venules in a ring-shaped area located between 0.5 and 1.0 disc diameter from the optic disc margin were identified (Figure 1). Computer software measured the calibre of these individual vessels, then combined them into 2 summary variables for the eye: the projected calibre size of the central retinal artery (central retinal artery equivalent [CRAE]), and the projected calibre size of the central retinal vein (central retinal vein equivalent [CRVE]), using formulas derived by Parr and Spears ([Parr and Spears, 1974a](#_ENREF_8), [b](#_ENREF_9)) and Hubbard ([Hubbard et al., 1999](#_ENREF_3)), with revision by Knudtson ([Knudtson et al., 2003](#_ENREF_5)). A retinal photograph was considered ungradable if eyes had <4 acceptable measurements of either vessel type. The intergrader and intragrader grading reliabilities were assessed using a random subsample of 100 photographs reviewed four weeks after the initial grading. The intra- and intergrader intraclass correlation coefficients ranged from 0.71 to 0.93. Retinal arteriolar and venular calibre are highly correlated, and to account for potential confounding we adjusted for the fellow vessel in multivariable analyses (i.e. adjustment for arteriolar calibre in analyses of venular calibre and vice versa) ([Liew et al., 2006](#_ENREF_6)).

**Table 1: Frailty criteria: adapted from Fried et al.(**[**Fried et al., 2001**](#_ENREF_2)**) and Önen et al.(**[**Onen et al., 2009**](#_ENREF_7)**)**

| **Criteria** | **Definition** | | | |
| --- | --- | --- | --- | --- |
| Unintentional weight loss | >10 pounds weight loss documented in last year or ≥5% of previous year’s body weight | | | |
| Low physical activity* | Participants answering 3 when asked whether their health limits vigorous activities such as running, lifting heavy objects  1= not at all, 2 = yes, limited a little or 3 = yes, limited a lot | | | |
| Exhaustion | Participants answering 2 or 3 to either one of two statements –  “How often have you felt that:”  a) Everything you did was an effort or  b) I could not ‘get going’  0 = rarely (<1 day), 1= some of the time (1-2 days),  2 = occasionally (3-4 days) or 3 = most of the time (5-7 days) | | | |
| Weak grip strength | Male BMI kg/m^2^  ≤24  24.1-26.0  26.1-28.0  >28 | Kg  ≤29  ≤30  ≤30  ≤32 | Female BMI kg/m^2^  ≤23  23.1-26.0  26.1-29  >29.0 | Kg  ≤17  ≤17.3  ≤18  ≤21 |
| Slow walking time | Male height (cm)  ≤173  >173 | Seconds  ≥7  ≥6 | Female height (cm)  ≤159  >159 | Seconds  ≥7  ≥6 |

*Estimation of physical activity adopted from Önen et al.([Onen et al., 2009](#_ENREF_7)); the estimation of physical activity described in the original phenotype used a weighted score of kilocalories expended

**References:**

Cesari, M., Kritchevsky, S.B., Penninx, B.W., Nicklas, B.J., Simonsick, E.M., Newman, A.B., Tylavsky, F.A., Brach, J.S., Satterfield, S., Bauer, D.C., Visser, M., Rubin, S.M., Harris, T.B., Pahor, M., 2005. Prognostic value of usual gait speed in well-functioning older people--results from the Health, Aging and Body Composition Study. J Am Geriatr Soc 53, 1675-1680.

Fried, L.P., Tangen, C.M., Walston, J., Newman, A.B., Hirsch, C., Gottdiener, J., Seeman, T., Tracy, R., Kop, W.J., Burke, G., McBurnie, M.A., Group, C.H.S.C.R., 2001. Frailty in older adults: evidence for a phenotype. J Gerontol A Biol Sci Med Sci 56, M146-156.

Hubbard, L.D., Brothers, R.J., King, W.N., Clegg, L.X., Klein, R., Cooper, L.S., Sharrett, A.R., Davis, M.D., Cai, J., 1999. Methods for evaluation of retinal microvascular abnormalities associated with hypertension/sclerosis in the Atherosclerosis Risk in Communities Study. Ophthalmology 106, 2269-2280.

Kirkwood, B.J., Hendicott, P.L., Read, S.A., Pesudovs, K., 2009. Repeatability and validity of lens densitometry measured with Scheimpflug imaging. J Cataract Refract Surg 35, 1210-1215.

Knudtson, M.D., Lee, K.E., Hubbard, L.D., Wong, T.Y., Klein, R., Klein, B.E., 2003. Revised formulas for summarizing retinal vessel diameters. Curr Eye Res 27, 143-149.

Liew, G., Wong, T.Y., Mitchell, P., Wang, J.J., 2006. Are narrower or wider retinal venules associated with incident hypertension? Hypertension 48, e10; author reply e11.

Onen, N.F., Agbebi, A., Shacham, E., Stamm, K.E., Onen, A.R., Overton, E.T., 2009. Frailty among HIV-infected persons in an urban outpatient care setting. J Infect 59, 346-352.

Parr, J.C., Spears, G.F., 1974a. General caliber of the retinal arteries expressed as the equivalent width of the central retinal artery. Am J Ophthalmol 77, 472-477.

Parr, J.C., Spears, G.F., 1974b. Mathematic relationships between the width of a retinal artery and the widths of its branches. Am J Ophthalmol 77, 478-483.

Wong, T.Y., Knudtson, M.D., Klein, R., Klein, B.E.K., Meuer, S.M., Hubbard, L.D., 2004. Computer-assisted measurement of retinal vessel diameters in the Beaver Dam Eye Study: methodology, correlation between eyes, and effect of refractive errors. Ophthalmology 111, 1183-1190.
