# Supplementary material for: Assessment of candidate ocular biomarkers of ageing in a South African adult population: Relationship with chronological age and systemic biomarkers
Source: Mech Ageing Dev. 2013 Jul;134(7-8):338–45. doi: 10.1016/j.mad.2013.05.002 (PMC3710972; doi:10.1016/j.mad.2013.05.002)
Supplement: Supplementary file 2 [file mmc2.docx]

**Table S1: Median values of candidate biomarkers by gender and age group**

**P-value for difference between genders**

|  |  | **Men** | | | **Women** | | | **P-value** |
| --- | --- | --- | --- | --- | --- | --- | --- | --- |
| **Measure** | **Age group, years** | **N** | **Median** | **IQR** | **N** | **Median** | **IQR** |  |
| **Systemic biomarkers** |  |  |  |  |  |  |  |  |
| Telomere length  Rel T/S | Overall | 60 | 1.04 | 0.86-1.29 | 190 | 1.09 | 0.91-1.32 | 0.62 |
|  | 30-39 | 26 | 1.07 | 0.93-1.29 | 90 | 1.13 | 0.94-1.30 |  |
|  | 40-49 | 25 | 1.15 | 0.92-1.30 | 55 | 1.10 | 0.94-1.34 |  |
|  | >50 | 9 | 0.96 | 0.57-1.17 | 45 | 0.99 | 0.88-1.29 |  |
| CDKN2A expression | Overall | 54 | 0.43 | 0.20-0.67 | 163 | 0.37 | 0.20-0.59 | 0.79 |
|  | 30-39 | 25 | 0.29 | 0.17-0.60 | 73 | 0.28 | 0.15-0.49 |  |
|  | 40-49 | 21 | 0.44 | 0.19-0.72 | 49 | 0.52 | 0.30-0.80 |  |
|  | >50 | 8 | 0.46 | 0.27-0.95 | 41 | 0.40 | 0.21-0.69 |  |
| **Lens density**  **Scale 0-100** |  |  |  |  |  |  |  |  |
| Linear | Overall | 59 | 9.8 | 9.2-10.8 | 187 | 9.8 | 9.2-10.8 | 0.89 |
|  | 30-39 | 26 | 9.3 | 9.0-9.7 | 91 | 9.3 | 8.9-9.6 |  |
|  | 40-49 | 24 | 9.9 | 9.5-11.1 | 54 | 10.3 | 9.8-11.0 |  |
|  | >50 | 9 | 11.5 | 10.9-12.4 | 42 | 11.3 | 10.7-12.11 |  |
| Peak | Overall | 59 | 17.7 | 15.4-19.3 | 187 | 18.2 | 15.4-20.8 | 0.39 |
|  | 30-39 | 26 | 17.2 | 13.6-19.2 | 91 | 16.2 | 14.8-18.7 |  |
|  | 40-49 | 24 | 18 | 15.6-19.0 | 54 | 18.8 | 15.7-20.1 |  |
|  | >50 | 9 | 20.3 | 16.8-23.4 | 42 | 22.4 | 19.6-24.8 |  |
| 3-D average | Overall | 59 | 9.8 | 9.1-10.7 | 187 | 9.8 | 9.1-10.9 | 0.77 |
|  | 30-39 | 26 | 9.1 | 8.8-9.6 | 91 | 9.2 | 8.8-9.6 |  |
|  | 40-49 | 24 | 9.9 | 9.5-10.8 | 54 | 10.2 | 9.7-11.1 |  |
|  | >50 | 9 | 11.6 | 10.7-12.7 | 42 | 11.4 | 10.8-12.6 |  |
| **Retinal vessel calibre μm** |  |  |  |  |  |  |  |  |
| CRAE | Overall | 59 | 161.49 | 148.11-173.6 | 184 | 160.95 | 150.95-172.59 | 0.91 |
|  | 30-39 | 26 | 167.03 | 156.15-175.63 | 91 | 160.81 | 151.01-171.43 |  |
|  | 40-49 | 25 | 159.12 | 140.14-173.3 | 54 | 158.08 | 146.40-169.72 |  |
|  | >50 | 8 | 158.9 | 152.67-168.5 | 39 | 167.90 | 155.58-177.18 |  |
| CRVE | Overall | 59 | 273.00 | 258.59-287.24 | 184 | 267.74 | 258.17-279.53 | 0.15 |
|  | 30-39 | 26 | 273.10 | 258.95-287.53 | 91 | 270.15 | 258.92-278.53 |  |
|  | 40-49 | 25 | 278.88 | 256.62-292.41 | 54 | 265.68 | 254.25-279.92 |  |
|  | >50 | 8 | 269.17 | 256.92-274.30 | 39 | 270.51 | 258.06-284.57 |  |
| **Endothelial cell parameters** |  |  |  |  |  |  |  |  |
| ECD | Overall | 59 | 2646 | 2461-2784 | 183 | 2582 | 2458-2812 | 0.85 |
|  | 30-39 | 25 | 2715 | 2546-2861 | 91 | 2602 | 2448-2894 |  |
|  | 40-49 | 25 | 2662 | 2315-2778 | 52 | 2650 | 2534-2788 |  |
|  | >50 | 9 | 2561 | 2211-2802 | 40 | 2526 | 2382-2747 |  |
| CV | Overall | 59 | 35 | 32-37 | 183 | 35 | 32-38 | 0.85 |
|  | 30-39 | 25 | 35 | 31-38 | 91 | 35 | 31-37 |  |
|  | 40-49 | 25 | 34 | 32-37 | 52 | 36 | 33-39 |  |
|  | >50 | 9 | 36 | 35-38 | 40 | 36 | 33-40 |  |
| Ex | Overall | 59 | 49 | 47-53 | 183 | 50 | 45-54 | 0.97 |
|  | 30-39 | 25 | 50 | 48-53 | 91 | 51 | 46-55 |  |
|  | 40-49 | 25 | 49 | 48-54 | 52 | 49 | 44-54 |  |
|  | >50 | 9 | 48 | 42-52 | 40 | 49 | 44-52 |  |
| **RNFL thickness, μm** |  |  |  |  |  |  |  |  |
| Average | Overall | 42 | 104 | 96-112 | 146 | 110 | 102-120 | 0.01 |
|  | 30-39 | 18 | 110 | 98-118 | 72 | 113 | 107-121 |  |
|  | 40-49 | 17 | 103 | 98-111 | 42 | 106 | 99-115 |  |
|  | >50 | 7 | 96 | 95-101 | 32 | 105 | 98-116 |  |
| Superior | Overall | 49 | 130 | 115-147 | 154 | 132 | 121-146 | 0.36 |
|  | 30-39 | 20 | 132 | 116-149 | 78 | 139 | 123-149 |  |
|  | 40-49 | 21 | 133 | 116-146 | 44 | 129 | 121-141 |  |
|  | >50 | 8 | 116 | 102-138 | 32 | 128 | 116-146 |  |
| Inferior | Overall | 48 | 131 | 111-143 | 154 | 140 | 127-154 | 0.0008 |
|  | 30-39 | 20 | 141 | 115-150 | 78 | 148 | 133-157 |  |
|  | 40-49 | 20 | 127 | 110-140 | 44 | 136 | 124-149 |  |
|  | >50 | 8 | 127 | 105-137 | 32 | 136 | 123-144 |  |
| Nasal | Overall | 49 | 86 | 63-101 | 154 | 89 | 76-103 | 0.19 |
|  | 30-39 | 20 | 97 | 69-109 | 78 | 85 | 71-102 |  |
|  | 40-49 | 21 | 84 | 61-94 | 44 | 91 | 75-107 |  |
|  | >50 | 8 | 85 | 51-86 | 32 | 88 | 81-102 |  |
| Temporal | Overall | 49 | 69 | 60-74 | 154 | 73 | 63-82 | 0.02 |
|  | 30-39 | 20 | 67 | 59-73 | 78 | 78 | 70-85 |  |
|  | 40-49 | 21 | 70 | 65-76 | 44 | 68 | 61-80 |  |
|  | >50 | 8 | 66 | 54-84 | 32 | 67 | 59-81 |  |
| **Frailty status** |  | **N** | **%** |  | **N** | **%** |  | **P-value** |
| Non-frail | Overall | 22 | 34.4 |  | 70 | 36.4 |  | 0.29 |
|  | 30-39 | 11 | 40.7 |  | 48 | 52.8 |  |  |
|  | 40-49 | 9 | 32.1 |  | 18 | 32.1 |  |  |
|  | >50 | 2 | 22.2 |  | 4 | 8.9 |  |  |
| Pre-frail | Overall | 39 | 60.9 |  | 91 | 47.4 |  | 0.28 |
|  | 30-39 | 16 | 59.2 |  | 40 | 44.0 |  |  |
|  | 40-49 | 17 | 60.7 |  | 28 | 50.0 |  |  |
|  | >50 | 6 | 66.7 |  | 23 | 51.1 |  |  |
| Frail | Overall | 3 | 4.7 |  | 31 | 16.2 |  | 0.47 |
|  | 30-39 | 0 | 0 |  | 3 | 3.3 |  |  |
|  | 40-49 | 2 | 7.1 |  | 10 | 17.9 |  |  |
|  | >50 | 1 | 11.1 |  | 18 | 40.0 |  |  |

**Table S2: Regression coefficients of biomarkers with chronological age in years**

| **Biomarker** | **N** | **Coefficient** | **R-squared** | **P-value** |
| --- | --- | --- | --- | --- |
| Telomere length | 250 | -0.0015 | 0.02 | 0.05 |
| CDKN2A | 217 | 0.006 | 0.02 | 0.02 |
|  |  |  |  |  |
| *Lens density* |  |  |  |  |
| Linear | 246 | 0.11 | 0.67 | <0.0001 |
| Peak | 246 | 0.30 | 0.25 | <0.0001 |
| Average 3D | 246 | 0.12 | 0.63 | <0.0001 |
|  |  |  |  |  |
| *Retinal vessel calibre* |  |  |  |  |
| Arteriolar diameter | 243 | 40-49 years: -4.45  >50 years: 4.15 | 0.03 | 0.02 |
| Venular diameter | 243 | -0.02 | 0.0001 | 0.86 |
|  |  |  |  |  |
| *Endothelial cell parameters*** |  |  |  |  |
| ECD | 242 | -5.40 | 0.04 | 0.003 |
| CV | 242 | 0.06 | 0.01 | 0.07 |
| Ex | 242 | -0.14 | 0.04 | 0.001 |
|  |  |  |  |  |
| *Retinal nerve fibre layer thickness* |  |  |  |  |
| Average | 188 | -0.39 | 0.07 | <0.0001 |
| Superior | 203 | -0.40 | 0.03 | 0.02 |
| Inferior | 202 | -0.51 | 0.04 | 0.003 |
| Nasal | 203 | -0.03 | 0.0001 | 0.88 |
| Temporal | 203 | -0.36 | 0.06 | 0.003 |

Linear regression models using age in years as a continuous variable in years or as a categorical variable for retinal vessel parameters

**ECD: endothelial cell density - lowest quartile denotes aged phenotype

CV: coefficient of variation – i.e. difference in cell shape; highest quartile denotes aged phenotype

Ex: Hexagonality index – i.e. proportion of cells that are hexagonal; lowest quartile denotes aged phenotype

**Table S3: Association of retinal nerve fibre layer with blood-based biomarkers**

| **RNFL Quartiles (μm)** |  |  |  |  |  |  |
| --- | --- | --- | --- | --- | --- | --- |
| Average |  |  |  |  |  |  |
| 1st  (65-101) | 49 | 1.06  (0.99-1.15) |  | 45 | 0.42  (0.33-0.54) |  |
| 2nd  (102-109) | 48 | 1.10  (1.02-1.18) |  | 44 | 0.28  (0.22-0.36) |  |
| 3rd  (110-117) | 45 | 1.11  (1.03-1.20) |  | 40 | 0.30  (0.24-0.39) |  |
| 4th  (118-149) | 46 | 1.07  (1.00-1.16) | 0.84 | 46 | 0.40  (0.31-0.51) | 0.07 |
| Superior |  |  |  |  |  |  |
| 1st  (61-118) | 48 | 1.02  (0.95-0.10) |  | 41 | 0.27  (0.21-0.35) |  |
| 2nd  (119-131) | 50 | 0.99  (0.94-1.09) |  | 43 | 0.39  (0.31-0.50) |  |
| 3rd  (132-146) | 53 | 1.11  (0.96-1.20) |  | 48 | 0.32  (0.25-0.40) |  |
| 4th  (147-199) | 46 | 1.11  (0.98-1.20) | P-trend 0.05 | 43 | 0.40  (0.32-0.52) | 0.09 |
| Inferior |  |  |  |  |  |  |
| 1st  (78-124) | 48 | 1.03  (0.95-1.11) |  | 41 | 0.33  (0.26-0.43) |  |
| 2nd  (125-138) | 51 | 1.07  (0.99-1.15) |  | 43 | 0.34  (0.27-0.44) |  |
| 3rd  (139-151) | 50 | 1.08  (1.00-1.17) |  | 48 | 0.33  (0.26-0.43) |  |
| 4th  (151-201) | 48 | 1.07  (0.99-1.16) | 0.80 | 43 | 0.36  (0.28-0.47) | 0.97 |
| Temporal |  |  |  |  |  |  |
| 1st  (38-62) | 47 | 1.04  (0.96-1.12) |  | 41 | 0.31  (0.24-0.40) |  |
| 2nd  (63-71) | 53 | 1.07  (1.00-1.15) |  | 51 | 0.32  (0.26-0.40) |  |
| 3rd  (72-81) | 48 | 1.10  (1.02-1.19) |  | 43 | 0.42  (0.33-0.53) |  |
| 4th  (82-131) | 49 | 1.04  (0.97-1.12) | 0.73 | 40 | 0.34  (0.26-0.43) | 0.33 |

Adjusted for age, gender, smoking, BMI, mean arterial blood pressure
