# Supplementary figures and images for: Assessment of candidate ocular biomarkers of ageing in a South African adult population: Relationship with chronological age and systemic biomarkers
Source: Mech Ageing Dev. 2013 Jul;134(7-8):338–45. doi: 10.1016/j.mad.2013.05.002 (PMC3710972; doi:10.1016/j.mad.2013.05.002)

**Supplementary Figures – See main manuscript for captions**

1d:


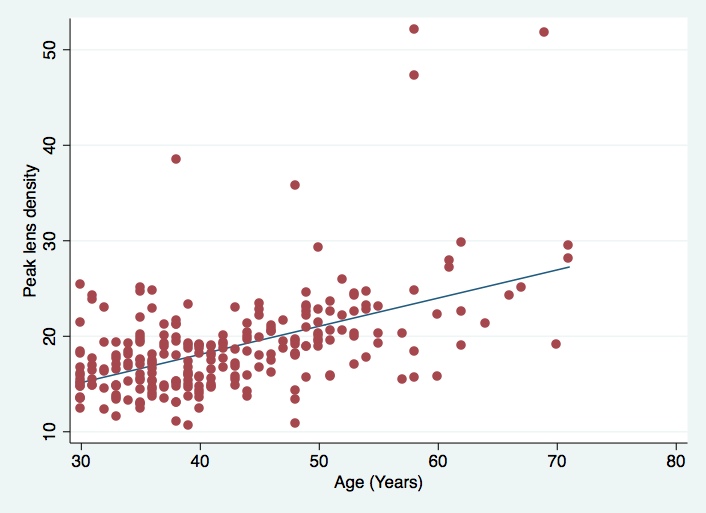


1e:


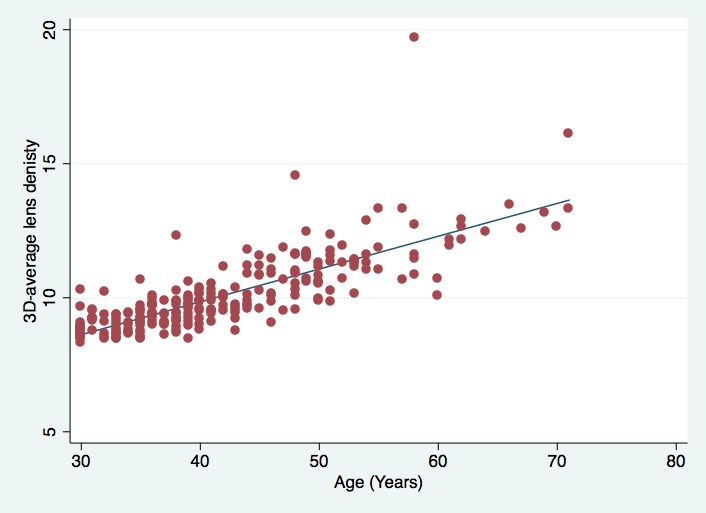


1f:


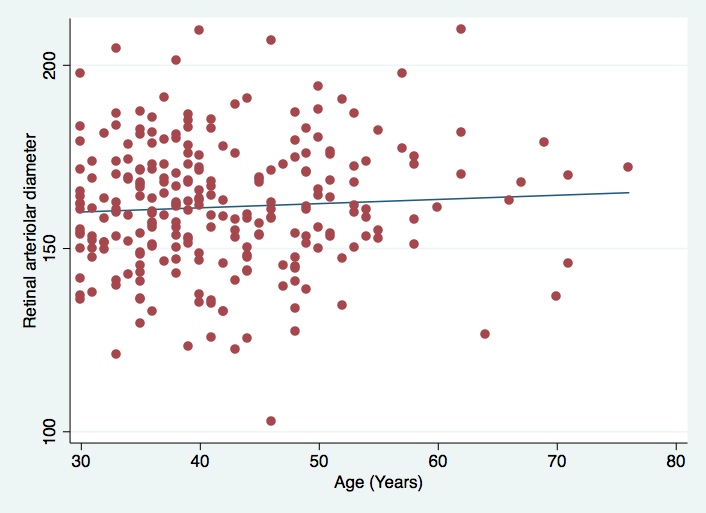


1g:


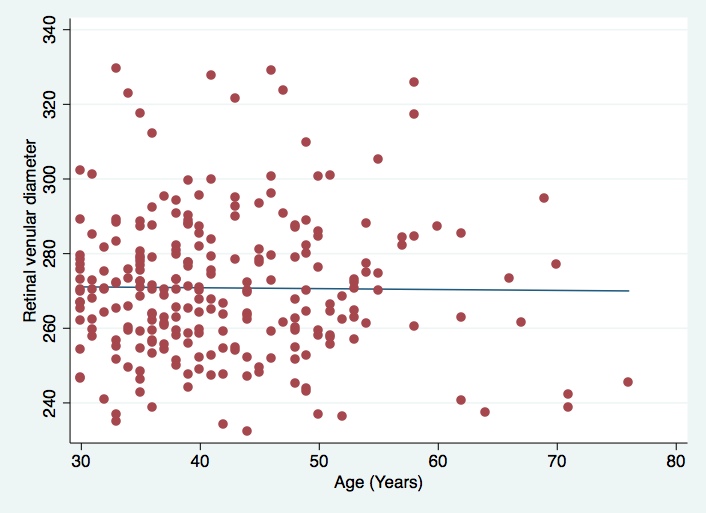


1h:


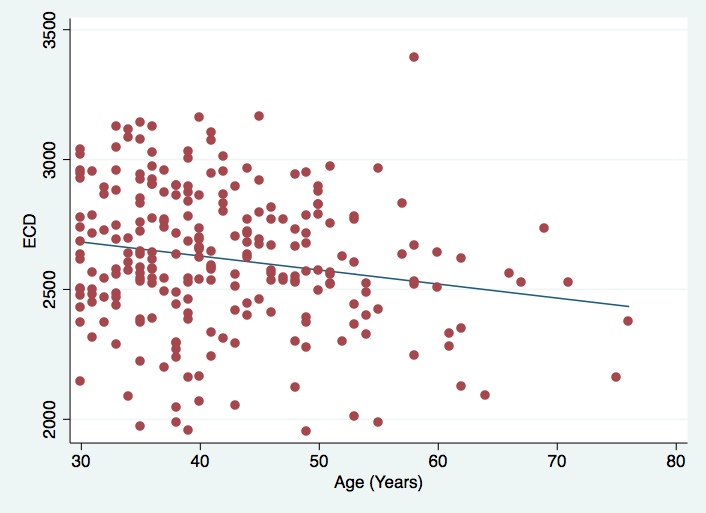


1i:


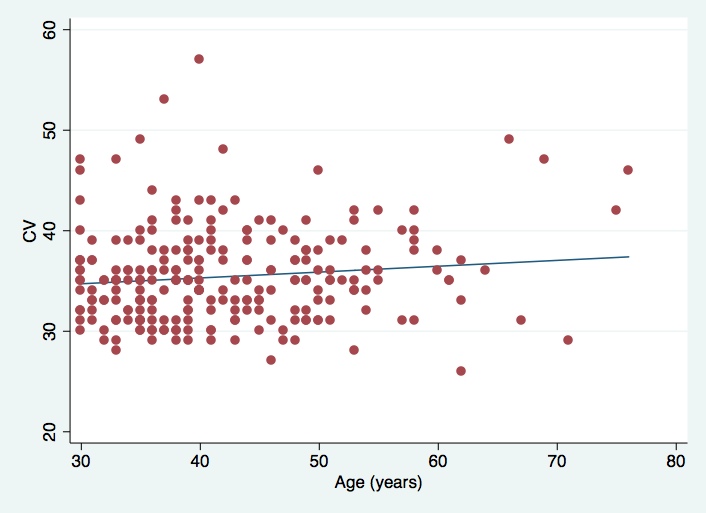


1j:


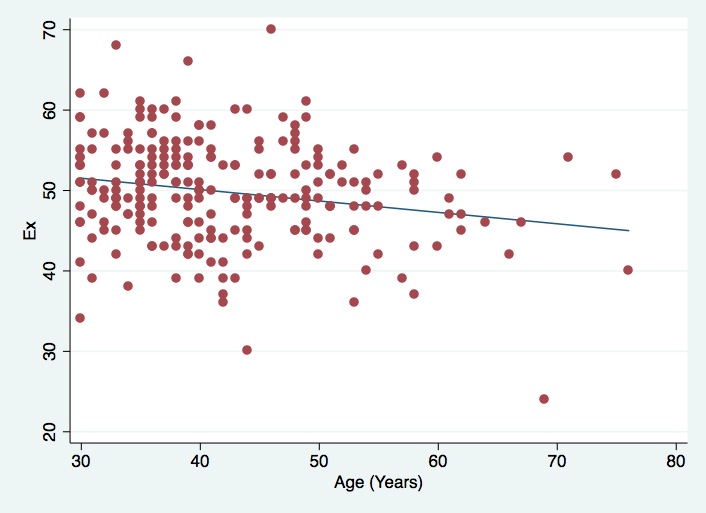


1k:


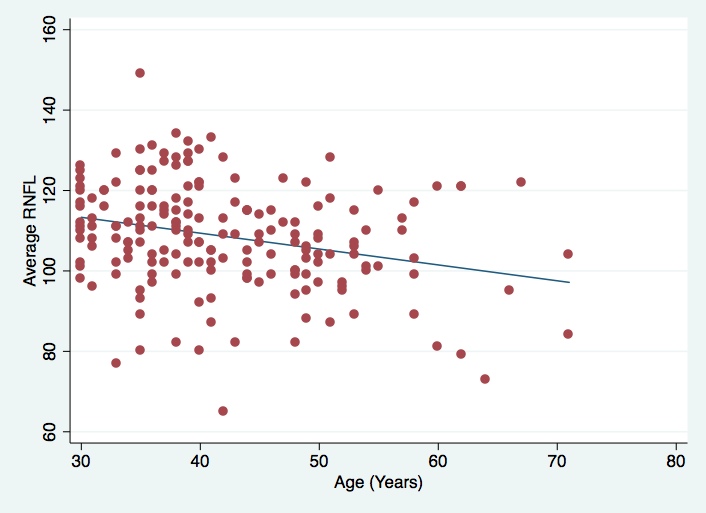


1l:


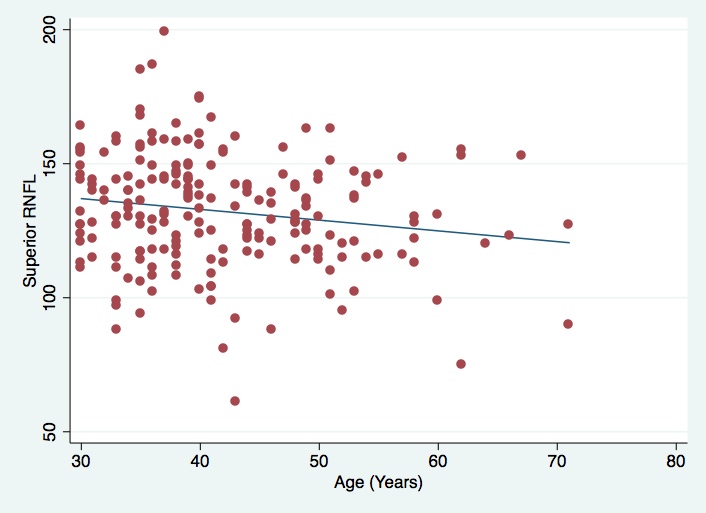


1m:


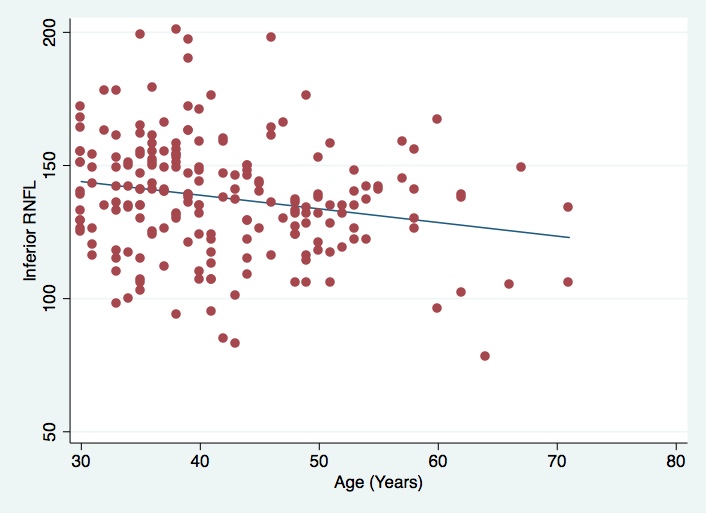


1n:


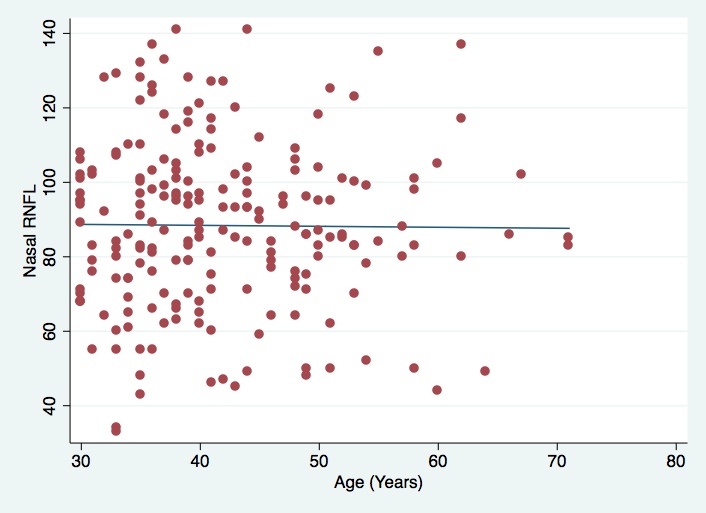


1o:


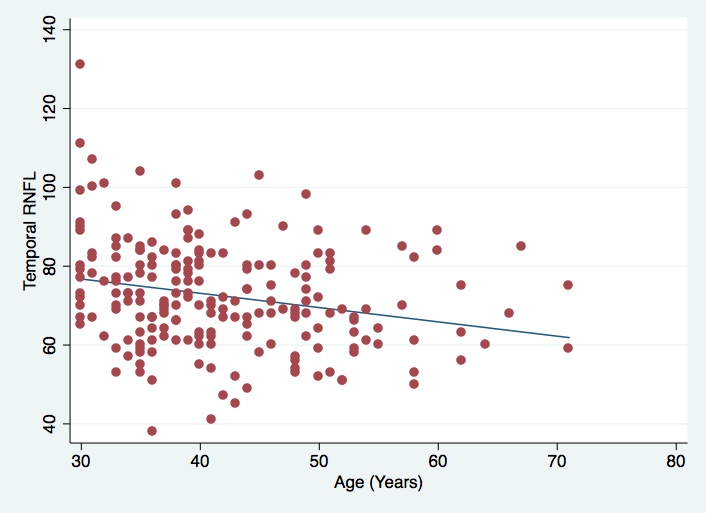

Supplement: Supplementary file 3 [file mmc3.docx]
